# Novel Symbiotic Genome-Scale Model Reveals *Wolbachia*'s Arboviral Pathogen Blocking Mechanism in Aedes aegypti

**DOI:** 10.1128/mBio.01563-21

**Published:** 2021-10-12

**Authors:** Natalia E. Jiménez, Ziomara P. Gerdtzen, Álvaro Olivera-Nappa, J. Cristian Salgado, Carlos Conca

**Affiliations:** a Centre for Biotechnology and Bioengineering (CeBiB), Department of Chemical Engineering, Biotechnology and Materials, University of Chile, Santiago, Chile; b Center for Mathematical Modeling (CMM), Department of Mathematical Engineering, University of Chile and UMI CNRS 2807, Santiago, Chile; c Fondap Center for Genome Regulation (Fondap 15090007), Universidad de Chile, Santiago, Chile; Korea Advanced Institute of Science and Technology

**Keywords:** *Wolbachia pipientis*, metabolic reconstruction, pathogen blocking, *Aedes aegypti*, genome-scale model

## Abstract

*Wolbachia* are endosymbiont bacteria known to infect arthropods causing different effects, such as cytoplasmic incompatibility and pathogen blocking in Aedes aegypti. Although several *Wolbachia* strains have been studied, there is little knowledge regarding the relationship between this bacterium and their hosts, particularly on their obligate endosymbiont nature and its pathogen blocking ability. Motivated by the potential applications on disease control, we developed a genome-scale model of two *Wolbachia* strains: *w*Mel and the strongest Dengue blocking strain known to date: *w*MelPop. The obtained metabolic reconstructions exhibit an energy metabolism relying mainly on amino acids and lipid transport to support cell growth that is consistent with altered lipid and cholesterol metabolism in *Wolbachia*-infected mosquitoes. The obtained metabolic reconstruction was then coupled with a reconstructed mosquito model to retrieve a symbiotic genome-scale model accounting for 1,636 genes and 6,408 reactions of the Aedes aegypti-*Wolbachia* interaction system. Simulation of an arboviral infection in the obtained novel symbiotic model represents a metabolic scenario characterized by pathogen blocking in higher titer *Wolbachia* strains, showing that pathogen blocking by *Wolbachia* infection is consistent with competition for lipid and amino acid resources between arbovirus and this endosymbiotic bacteria.

## INTRODUCTION

*Wolbachia* are obligate intracellular symbionts, members of the *Rickettsiales* group, known to infect over 65% of all insects species, mainly arthropods, developing diverse interactions with their hosts, such as supplementation with vitamins (1, 2), cytoplasmic incompatibility (3, 4), pathogenic interactions (5, 6), and pathogen blocking (7, 8), among others.

Due to their pathogen-blocking abilities, these endosymbionts have been of special interests in disease control. In particular, the nonpathogenic strain *w*Mel, originally found in Drosophila melanogaster, has been used for infecting Aedes aegypti, obtaining mosquitoes that exhibited almost no transmission of Dengue and Zika viruses (7, 8). However, the mechanisms behind *Wolbachia*-mediated pathogen blocking have not been fully characterized. Two main hypotheses have been proposed for explaining this phenomenon, one associated with an improved immunological response as a consequence of *Wolbachia* infection and the other one related to competition for host cell resources in the synthesis of key building blocks for this endosymbiont and arboviruses (9).

We propose that a thorough analysis of *Wolbachia*'s metabolism could unveil key aspects of the metabolic relationship between this endosymbiont and its host and how their interactions influence the pathogen-blocking response in Aedes aegypti. This representation of *Wolbachia* metabolic capabilities will be given by genome-scale models (GSMs). Genome-scale models have emerged as a powerful tool for studying cellular metabolism based on their genome annotation (10). In particular, recent applications of GSMs for studying endosymbiotic organisms in insects have shown the potential of this approach (11, 12), these analysis focused on the interactions between endosymbionts found in *Cinara cedri* (12) and Bemisia tabaci (11) finding that the coexistence of endosymbionts leads to an additional reduction of genetic information and a fragile metabolic network (12). However, none of these efforts has explicitly considered the whole metabolism of the host insect and how it is affected by its interaction with these endosymbiotic bacteria using a GSM approach, but only the immediate extracellular environment of the bacteria.

As a result of its adaptation to depend on another organism for its survival, Wolbachia pipientis has a reduced genome size compared to similar nonendosymbiotic organisms. This leads to a small and rather incomplete metabolic network, as has been observed previously for other endosymbiotic bacteria (11–14). We hypothesized that the analysis of the metabolic gaps in the curation stage of the metabolic reconstruction could reveal potential candidates that explain the mutualistic relationship between *Wolbachia* and Aedes aegypti.

In this work, we present three novel metabolic reconstructions developed for the study of Wolbachia pipientis and Aedes aegypti endosymbiosis from a systems biology perspective: a genome-scale model for *W. pipientis* and A. aegypti and the first model that represents the endosymbiosis between these two organisms. We propose that the metabolic analysis of the interactions between these organisms holds key features for explaining *Wolbachia*'s mediated pathogen blocking and that this blocking effect is a direct consequence of competition for the host resources.

## RESULTS AND DISCUSSION

### Metabolic reconstruction for the symbiotic bacteria *Wolbachia*.

Two Wolbachia pipientis strains were selected for this work based on data availability and known pathogen-blocking capabilities: the Drosophila melanogaster endosymbionts *w*Mel and *w*MelPop. The obtained genome-scale models were used as a base to reconstruct the Wolbachia pipientis genome-scale model (iNJ644). This model includes 644 genes and 790 reactions, of which 220 are orphan reactions predicted to be present in the model despite the fact that some genes associated with this reaction are absent from *Wolbachia* genomes (13). These reactions are added to ensure that a functional model, capable of representing the synthesis of all the required components for cell growth, is obtained in the reconstruction process (10).

Validation is performed to test if the obtained models can accurately represent reported and experimental data available for this endosymbiont. Information regarding *Wolbachia* (15, 16) and *Rickettsia*'s metabolism (14) was gathered from the literature to compare metabolic features predicted by this metabolic reconstruction with the ones reported for these organisms (Table 1).

**TABLE 1:** Wolbachia pipientis genome-scale model (iNJ644) validation^*a*^

No.	Test	Strain	Result
1	Amino acid transport (Pro, Asp/Glu, Ala)	*w*Mel	+
2	Amino acid metabolism (Gly, Glu, Gln, Pro, Ser, Thr)	*w*Mel	+
3	Inability to produce LPS	*w*Mel	+
4	Complete pentose phosphate pathway	*w*Mel	+
5	Absence of ADP-ATP exchanger protein	*w*Mel	−
6	Threonine degradation pathway	*w*Mel	+
7	Riboflavin biosynthesis	*w*Mel	+
8	Complete TCA cycle	*w*Mel	+
9	Glycolysis starting from fructose 1,6 BP	*w*Mel	+
10	Lethal inhibition of *MurA*	*w*Bm	++
11	Nonlethal inhibition of *DdlA*	*w*Bm	−−

a Validation tests based on metabolic features reported for Wolbachia pipientis *w*Mel and *w*Bm (Brugia malayi). Present metabolic feature, +; absent metabolic feature, −; consistent with experimental observation, ++; inconsistent with experimental observation, −−.

We tested if *Wolbachia*'s preliminary model includes metabolic pathways previously reported to be present on Wolbachia pipientis, such as glycolysis starting from fructose 1,3 bisphosphate, a complete pentose phosphate pathway, TCA (tricarboxylic acid) cycle, an active amino acid metabolism, including transport of amino acids (proline, aspartate, glutamate and alanine), and catabolism of glycine, glutamate, glutamine, proline, serine, and threonine; the inability to produce lipopolysaccharides; and the absence of an ADP-ATP exchanger protein (15). Our analysis of the obtained metabolic network supports these affirmations, finding a complete glycolysis from fructose-6P toward phosphoenolpyruvate and a partially complete TCA cycle. The peptidoglycan synthesis pathway in *Wolbachia* and Chlamydia has been reported to be functional while growing inside their hosts. Two key points of this metabolic pathway were tested based on studies in peptidoglycan synthesis in *Wolbachia*, finding that inhibition of the first step of peptidoglycan synthesis catalyzed by the *murA* (UDP-*N*-acetylglucosamine 1-carbovinyltransferase) gene is lethal in *Wolbachia w*Bm infecting C6/36 cells (16). Additionally, they tested the inhibition of *Ddla* (d-alanine d-alanine ligase A) by d-cycloserine, finding that it does not have a negative effect on lipid II biosynthesis in *Wolbachia* cells.

Our *Wolbachia* reconstruction can replicate the lethal effect of phosphomycin *in silico*. The effect of high concentrations of this inhibitor is represented as a gene knockout, resulting in no cell growth in the performed simulations. iNJ644 knockout analysis predicts that inhibition of *ddlA* by d-cycloserine produces a nongrowth phenotype. *ddlA* is associated with the conversion of two alanines into one alanine dipeptide (Ala-Ala), which is a crucial step in lipid II synthesis. An additional blast search (coverage above 85% and E-values lower than 0.001) showed that the *Wolbachia* genome includes genes with high homology to the Ala-Ala transporters found in Escherichia coli, suggesting that *Wolbachia* could get this metabolite from its host to be consistent with experimental data (17, 18). As a consequence, transport of the alanine dipeptide was added to the *Wolbachia* metabolic reconstruction. A final curated model was obtained and analyzed to find metabolic candidates that explain the obligate intracellular character of this bacteria.

### Genome-scale model for Aedes aegypti.

To identify the key metabolic features that link *Wolbachia*'s metabolism with its host's, the metabolic gaps present in *Wolbachia*'s metabolic network were studied. To analyze *Wolbachia*'s pathway gaps in their metabolic context, we reconstructed a metabolic model for Aedes aegypti using an ortholog-based approach. Ortholog search between Aedes aegypti and Homo sapiens retrieved 5,327 ortholog groups between both organisms. This information was complemented with the gene associations present in the human metabolic reconstruction Recon 2.2 (19) to obtain the first Aedes aegypti metabolic model. This metabolic reconstruction accounts for 991 genes associated with 2,735 reactions and was then curated to include all the specific metabolic features reported for A. aegypti.

Since a human metabolic reconstruction was used as a template, known insect and A. aegypti metabolic features were added prior to validation of the obtained model. In particular, *Diptera* and specifically A. aegypti insects are unable to synthesize sterols from acetate (20, 21); hence, they acquire cholesterol from their diet (22). Particularly in Aedes aegypti, cholesterol transport is mediated by sterol carrier protein 2 (SCP2) (23–25). Overexpression of SCP2 has been found to increase incorporation of cholesterol (26), while its knockdown has led to a reduced uptake of cholesterol in female mosquito adults (27). Addition of SCP2 cholesterol transport is associated with the gene identifiers AAEL026044 and AAEL025252 (27, 28).

Additionally, a mosquito-specific pathway for urea disposal proposed by Scaraffia et al. (29), also known as the ureide pathway, was integrated into the mosquito metabolic reconstruction. It considers a series of reactions in which uric acid is transformed into allantoin by urate oxidase (UO), to allantoic acid by allantoinase (ALN), and to ureidoglycolate by alllantoicase (ALLC) (29). This pathway considers a final spontaneous reaction in which ureidoglycolate is transformed to glyoxylate and urea (urease) (5).

In *Diptera*, thioredoxin, as opposed to glutathione, is used as a redox buffer. Particularly for Anopheles gambiae, it has been reported that alternative splicing of a thioredoxin reductase gene (*trdR*) leads to mitochondrial and cytoplasmic variants that keep redox homeostasis in this organism (30). Based on the metabolic requirements of Aedes aegypti, a cytoplasmic variant of the *trdR* gene was added.

Experimental data regarding A. aegypti metabolism, growth media, and gene deletions were retrieved from the literature. Validation of this curated model was performed by simulating previous culture conditions in mosquito cell lines, metabolic observations, and gene knockdowns represented in the obtained reconstruction (Table 2). We tested if the obtained mosquito model is able to replicate lipid and sterol usage in Aedes aegypti cells (31), finding that growth in cholesterol, phosphatidylcholine, and phosphatidylethanolamine is accurately represented by this metabolic reconstruction, as is the use of palmitate, stereate, and oleate for sustaining cellular growth (31).

**TABLE 2:** Validation of the Aedes aegypti model^*a*^

Test	Result
Medium conditions	
Growth in cholesterol, phosphatidylcholine, and phosphatidylethanolamine	++
Unable to grow on palmitic acid, stearic acid, or oleic acids	++
Growth in ergosterol, zymosterol	−
Growth in sphingomyelin, β-carotene, or α-tocopherol acetate	−
Growth in proline as energy substrate	++
Gene deletions	
Sterol carrier protein (AeSCP2) knockdown resulted in higher mortality	++
Thioredoxin reductase (Trdx1) is necessary for survival	++
Inactivation of glutamine synthase (AeGS) is lethal	++
Blood fed mosquitoes with alanine transferase (ALT) is not lethal	++
Knockdown of xanthine dehydrogenase (XDH-1) is lethal to blood-fed mosquitoes	−
Arginase (AR) silencing is not lethal	++
Urate oxidase (UO) silencing is not lethal	++
AR and urate oxidase (UP) silencing is not lethal	++
Nitric oxide synthase silencing is not lethal	++

a Validation tests based on metabolic features reported for *Aedes aegytpi*. Consistent with experimental observation, ++; inconsistent with experimental observation, −.

The obtained metabolic reconstruction is unable to represent cellular growth based on ergosterol and zymosterol as lipid sources despite the fact that this has been extensively reported for this organism (31–33) and for the mosquito Culex pipiens (34). An analysis of the sterol pathway and search of similar sequences revealed no potential candidates in the Aedes aegypti genome that could lead to metabolization of these alternate sterol sources toward cholesterol and, hence, cell growth. On the other hand, this mosquito genome-scale model is able to represent cellular maintenance using proline as an energy source. The obtained cell growth is slower than the one obtained for glucose or trehalose as carbon sources but higher than the one reported with a lack of sterols in the media, which is consistent with proline being used only in extreme nutrient deprivation cases in mosquito flight response (35).

Although there are several studies where gene deletions or silencing have been used as a means of controlling mosquito population, most of these targets are associated with cellular processes that cannot be represented by a metabolic reconstruction, e.g., olfactory sensors (36), blood feeding (37), reproduction (38), or apoptosis (39–43), among others (44–49).

Urea formation in Aedes aegypti is achieved by two mechanisms: argininolysis and uricolysis. Gene silencing of arginase, urate oxidase, and nitric oxide synthase was revealed not to be lethal in this organism, consistent with gene deletion simulations (50). However, despite the fact that xanthine dehydrogenase 1 (XDH1) deletion has been proven lethal in blood-fed mosquitoes (51), gene deletion simulations for XDH1 and XDH2 were not able to replicate this behavior *in silico*. Since it has also been reported that this deletion had no effect on sugar-fed mosquitoes (51), we believe that there should be an unidentified process, currently not represented in our model, associated with blood metabolization where XDH1 is essential.

On the other hand, the obtained metabolic reconstruction can replicate the lethal effect of sterol carrier protein (*AeSCP2*) knockdown in Aedes aegypti mosquitoes (23, 52) and the essentiality of thioredoxin reductase in Anopheles gambiae (30) and of glutamine synthase in the mosquito-derived cell line C6/36 (53, 54).

Overall our metabolic model was able to replicate nearly 80% (11/14) of the tested conditions in which gene deletions were associated with metabolic functions that are represented in genome-scale models. The obtained model was subsequently coupled with our Wolbachia pipientis metabolic reconstruction to shed light on the metabolic processes associated with bacterial and insect symbiosis. Both the presented models and their curation process are described in detail in file S1 at https://github.com/natJimenez/symbioticModelAnalysis.

### A novel symbiotic model approach for studying *Wolbachia*-mosquito metabolic interactions.

**FIG 1:**
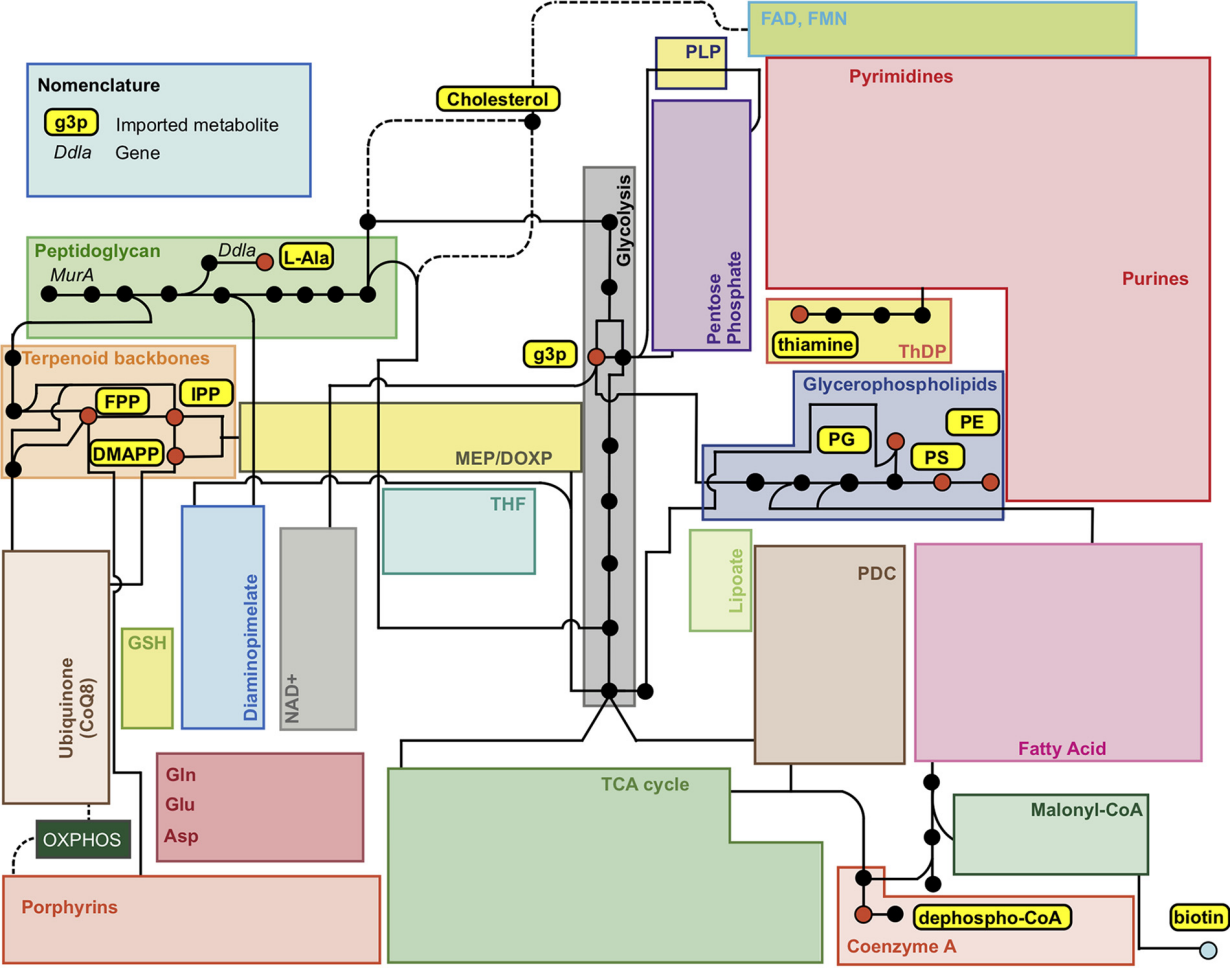
Wolbachia pipientis metabolism and its interactions with *Aedes aegytpi.* Key interaction points with Aedes aegypti are highlighted in red, complete metabolic pathways are represented as empty boxes, and dotted lines represent connections between metabolites with intermediate steps lumped. Only connections relevant to metabolic pathways of interest are presented. Adapted from reference 14 with permission. PLP, pyridoxal 5′-phosphate; ThDP, thiamine diphosphate; PDC, pyruvate dehydrogenase complex; TCA, tricarboxylic acid; MEP/DOXP, mevalonate-independent 2-C-methyl-d-erythritol 4-phosphate/1-deoxy-d-xylulose 5-phosphate pathway; THF, tetrahydrofolate; GSH, glutathione; OXPHOS, oxidative phosphorylation; g3p, glycerate 3 phosphate; PE, phosphatidylethanolamine; PS, phosphatidylserine; PG, phosphatidylglycerol; FPP, farnesyl diphosphate; IPP, isopentenyl phosphate; DMAPP, dimethylallyl pyrophosphate.

A symbiotic model was obtained by integration of the *Wolbachia* and Aedes aegypti metabolic model. Further curation was performed to connect *Wolbachia* precursors to the mosquito metabolic network (Fig. 1). We analyzed each metabolic requirement and its association with metabolic gaps in the *Wolbachia* model. Flux balance analysis (FBA) simulations showed that this endosymbiont requires the import of isopentenyl diphosphate (IPP) for biomass synthesis. These predictions are consistent with metabolite exchange predictions made for *Rickettsia* (14), obligate intracellular bacteria that belong to the same order as *Wolbachia*, where uptake of these metabolites is predicted to be essential for peptidoglycan and lipopolysaccharide biosynthesis (55).

*Wolbachia*'s metabolic model includes the exchange of metabolites that are either absent from the mosquito metabolic network or cannot be produced by Aedes aegypti in order to be retrieved by *Wolbachia*. In particular, lanosterol was predicted to be imported by this endosymbiont, but it is generally absent from the Aedes aegypti intracellular environment unless directly consumed by the mosquito. This metabolite is required by this symbiotic bacteria for farnesyl diphosphate (FPP) synthesis, a compound known to be imported in *Rickettsia* (14); hence, an additional FPP transport reaction was added to the *Wolbachia* model. Additionally, the lipid A synthesis pathway is completely absent from our *Wolbachia* metabolic reconstruction, as was previously reported for *w*Mel (15). Based on this information, we suggest that *Wolbachia* uses cholesterol instead of lipid A for its cell wall composition, as has been reported for other closely related endosymbiotic bacteria (56).

### Gap analysis of the symbiotic model unveils new features in *Wolbachia*'s lipid metabolism.

**FIG 2:**
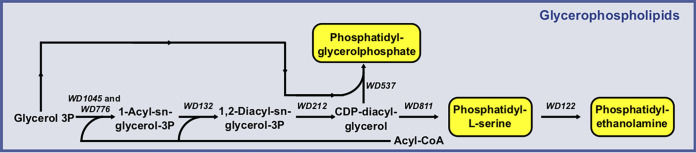
Wolbachia pipientis glycerophospholipid metabolism. *Wolbachia* can synthesize phosphatidylglycerolphosphate, phosphatidylserine, and phosphatidylethanolamine.

The *Wolbachia* genome-scale model includes reactions that allow synthesis of phosphatidylglycerol, phosphatidylserine, and phosphatidylethanolamine from acyl-coenzyme A (CoA), unlike what has been published previously for *w*Mel (15) (Fig. 2). However, the metabolic representation does not allow the synthesis of other membrane components, such as phosphatidylcholine and cardiolipin, making *Wolbachia* highly dependent on the host's intracellular membranes for cell growth.

Based on its association with intracellular membranes (57, 58), phospholipid import in the *Wolbachia* model was represented as a transport reaction that preserves the stoichiometric coefficients of the components present in the mosquito membrane. An analysis of A. aegypti and *W. pipientis* membrane composition stoichiometry showed that there is a surplus of cholesterol in *Wolbachia* that is not required for membrane synthesis. We speculate that this excess of cholesterol is drained in the form of the lipid droplets observed in *Wolbachia*-infected A. aegypti cells and that this imbalance is associated with the reported cholesterol-altered metabolism observed in mosquito-infected cells (59–61).

### *Wolbachia* symbiosis affects amino acid and cholesterol metabolism in A. aegypti.

*Wolbachia* and A. aegypti interaction was explored by analyzing 20,000 feasible solutions of the obtained model without imposing an optimality criterion, as illustrated in file S3 at https://github.com/natJimenez/symbioticModelAnalysis. Additionally, we analyzed the Pareto front (dashed lines), where the optimal use of resources toward *Wolbachia* and mosquito biomass synthesis is presented. These results evidence that, in an optimal distribution of resources, there is a trade-off between the growth rates of both organisms. This can be derived from the negative slope of the optimal use of the resource curve (Pareto front) for cell growth in this symbiotic system. Since the value of this slope is −2.4 [μ_Aedes aegypti_/μ*_Wolbachia_*], producing a mosquito cell requires more nutrients than producing a *Wolbachia* cell. For instance, in the scenario of optimal use of resources, a 0.04 mosquito cell growth rate is associated with nearly double this value for *Wolbachia* growth (file S3 at the GitHub link above).

The sampled fluxes exhibit a distribution with a stable state where low mosquito growth rates and a nonzero minimum *Wolbachia* growth rate are observed, given by *Wolbachia* riboflavin supplementation to A. aegypti. FBA simulations in a riboflavin-deprived environment show that although riboflavin supplementation is essential, higher growth rates of *Wolbachia* result in nutrient depletion and decreased mosquito biomass synthesis. The obtained results show that most of the feasible solutions were obtained around duplication times of 100 h for *Wolbachia* and between 0 and 400 h for Aedes aegypti. By analyzing duplication times of each organism (file S3 at https://github.com/natJimenez/symbioticModelAnalysis), we estimate that there is a median of 3.45 *Wolbachia* cells per mosquito. Given that quantification of *Wolbachia* density inside the mosquito cell line Aa23 was estimated as between 300 and 1,200 bacteria per mosquito cell (60), we propose that *Wolbachia* pressure for remaining in A. aegypti is mainly due to regulatory processes rather than metabolic interactions.

**FIG 3:**
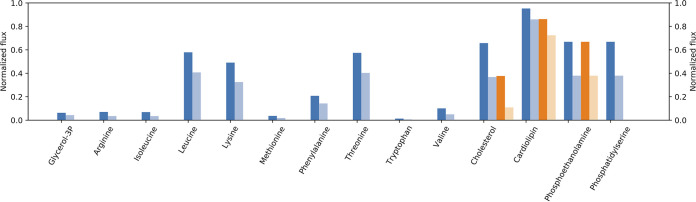
Wolbachia pipientis and Aedes aegypti metabolic interactions. Flux balance analysis simulations are performed considering variations in the ratio between *Wolbachia* and its host based on values obtained by sampling of the symbiotic model (file S3 at the GitHub link in the article text above). Carbon sources, amino acids, and lipids consumed by higher (dark blue) and lower (light blue) infecting *Wolbachia* densities. Lipids and lipid droplets are represented in dark orange and light orange, respectively.

Results show that *Wolbachia* relies mainly on amino acids for supporting its cell growth (Fig. 3); these amino acids are transported into the mosquito-*Wolbachia* endosymbiotic system and then consumed by *Wolbachia*. Tryptophan, valine, methionine, and leucine in particular have been reported as crucial for remediating low fecundity in *Wolbachia*-infected mosquitoes due to host competition for amino acids (62). Additionally, it has been reported that the presence of *Wolbachia* can reduce total cholesterol levels in mosquitoes up to 25%, which is consistent with the 20% total cholesterol consumed obtained in flux balance analysis simulations (Fig. 3).

The obtained results suggest that at least 10% of the total cholesterol present in mosquito cells is in the form of lipid droplets. This value is lower than the 25% *Wolbachia*-induced reduction of total cholesterol reported in Aedes aegypti mosquitoes (62) and the nearly 10% reduction of total cholesterol observed in the mosquito-derived cell line Aag2 infected with *w*MelPop (59). This is in agreement with the composition of mosquito cells considered in this study being closer to the one reported for mosquito-derived cell lines than to the variety of cell compositions found in whole insects.

Geoghegan et al. (59) studied Aedes aegypti's response to *Wolbachia* infection by analyzing proteomic data of Aag2 cells, showing that they exhibit an altered cholesterol metabolism similar to the responses associated with Niemann-Pick disease. We hypothesize that this alteration of sterol homeostasis could be triggered by cholesterol accumulation due to metabolites transported but not required for *Wolbachia* membrane synthesis. Elevation of esterified cholesterol has been associated with *Wolbachia* infection (59, 60, 62). Since our results show that these intracellular bacteria do not have genes known to interact with cholesterol- or sterol-derived metabolites, we propose that esterification is performed by Aedes aegypti rather than *Wolbachia* for sterols to be stored as lipid droplets. This would be the initial response of the metabolic cascade associated with intracellular cholesterol accumulation, which is followed by downregulation of LDL receptor and fatty acid synthase (59).

### *Wolbachia*'s pathogen blocking explained as competition for limited resources.

One of the main features of interest in *Wolbachia*-infected mosquitoes is pathogen blocking. Several hypotheses have been proposed for explaining the underlying mechanisms resulting in protection against arboviral infection granted by *Wolbachia* infection. Some of these mechanisms are associated with an immunological boost in mosquito cells due to the presence of this endosymbiont, and others are related to competition for host cell resources. Recent findings have shifted the interest toward the latter, with amino acids and lipids being the proposed bottlenecks for viral replication (59, 62, 63).

**FIG 4:**
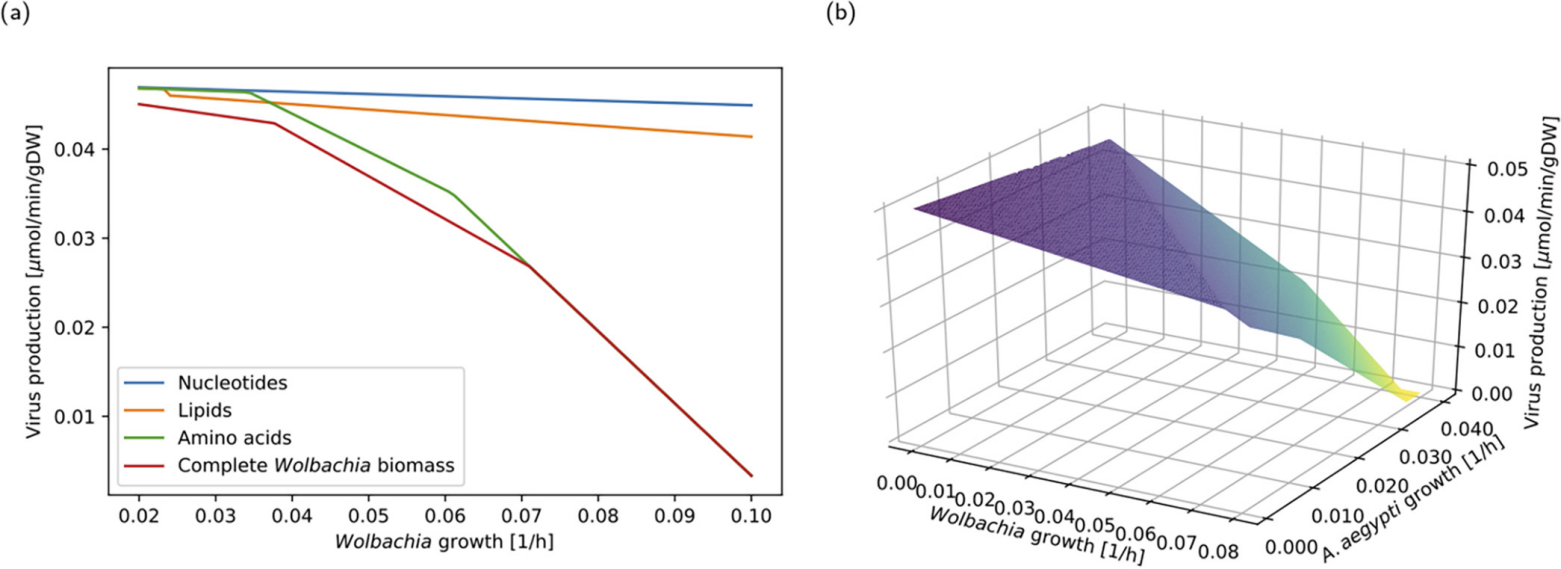
*Wolbachia*-mediated pathogen blocking: amino acid and lipid composition was derived from ZIKV and the West Nile virus. (a) Determination of key *Wolbachia* components in the pathogen-blocking phenotype. Simulations used different *Wolbachia* compositions comprising the nucleotide, amino acid, lipid fraction, and its effects on viral reproduction. (b) FBA simulations were performed using maximization of virus synthesis as their objective function, showing that higher Wolbachia pipientis densities are detrimental for viral synthesis.

In this work, we propose that genome-scale models are suitable for exploring the hypothesis that can be directly represented by chemical reactions, such as competition for host resources. Viral replication is represented in the endosymbiotic model as a metabolic reaction comprising the RNA, amino acid, and lipid composition of the Dengue virus (DENV) (64, 65). Flux balance analysis was performed to simulate viral infection in scenarios characterized by different *Wolbachia* and A. aegypti growth rates (Fig. 4). The obtained results indicate that this system shows pathogen blocking at high *Wolbachia* cellular densities, consistent with previously reported studies on pathogen blocking by 10 different *Wolbachia* strains, where higher titers were consistently found to exhibit stronger pathogen-blocking properties (66).

Additionally, we tested which component of *Wolbachia*'s biomass has a higher influence on pathogen blocking. Different artificial *Wolbachia* biomass compositions, including only amino acids, nucleotides, or lipids, were simulated in order to test which component is critical for pathogen blocking (Fig. 4), showing that amino acids are crucial for this phenotype. Amino acid depletion has also been linked to host cell responses to viral infections in the absence of *Wolbachia* infection. It has been reported that phosphorylation of eukaryotic initiation factor 2 alpha (eIF2α) as a consequence of a diminished amino acid pool leads to viral replication arrest (67). This increased demand for protein precursors contributes to the observed unfolded protein response (UPR) in *wMelPop*-infected organisms and is consistent with *Wolbachia*'s location near the endoplasmic reticulum in order to ensure a constant supply of amino acids to support its energy metabolism (68).

Recently this fact has been debated, since Fattouh et al. (58) found no UPR associated with *Wolbachia w*Mel infection, stating that this response is associated with the pathogenic behavior of wMelPop rather than *Wolbachia* itself. A previous analysis of metabolic networks for *w*Mel and *w*MelPop found that both strains are almost identical (55); in this approach, pathogenicity is represented by higher biomass production. Consistent with this, higher titers of *Wolbachia* are associated with a stronger pathogen-blocking phenotype.

Recently, Koh et al. (69) compared perturbation on lipid profiles of mosquitoes infected with DENV and *w*Mel, finding that the overlap between them was not enough to suggest direct competition for lipids in a pathogen-blocking scenario. Instead, they proposed that Wolbachia pipientis perturbs lipid metabolism in a way that is detrimental for viral synthesis. Based on their results, they performed knockdown of key genes in lipid metabolism, particularly cardiolipin synthase (CRLS), which had negative effects on both bacterial growth and viral production.

A comparison between Wolbachia pipientis, Aedes aegypti, and DENV lipid composition in our model shows that although *Wolbachia* and DENV share requirements for phosphatidylserine and phosphatidylethanolamine, most of their compositions differ. Even so, *Wolbachia* can synthesize both of these phospholipids from glycerol-3P (Fig. 2).

Koh et al. found two lipid classes that were enriched in a DENV infection but depleted in a dual *Wolbachia*-DENV infection: sphingomyelin and cardiolipin. In our model, sphingomyelin is considered part of the lipid fraction of DENV, which is synthesized from serine and palmitoyl-CoA in Aedes aegypti. Since sphingomyelin is not required for biomass synthesis for both mosquito and *Wolbachia*, an enrichment of this metabolite in a DENV infection scenario is consistent with our model.

Cardiolipin, on the other hand, is part of both Wolbachia pipientis and A. aegypti composition but not of the DENV lipid fraction. Their enrichment on the DENV infection scenario is explained as a mechanism to stop apoptosis instead of being directly destined for viral production, consistent with our analysis of viral composition. Depletion of cardiolipin in a dual infection could be explained by the inability of Wolbachia pipientis to synthesize this lipid, posing an additional sink in this scenario that is detrimental for viral synthesis.

Arboviral replication is a complex process that requires hijacking several metabolic processes in the mosquito cell, which alters membrane composition, drains intracellular nucleotides and energy, and subsequently modifies vesicle formation to ensure viral secretion (9). In particular, cholesterol has been reported to be of extreme importance in pathogen blocking (59, 61). Caragata et al. (61) showed that in D. melanogaster, higher dietary cholesterol resulted in reduced *Wolbachia*-mediated pathogen blocking against *Drosophila* C virus (DCV). In Aedes aegypti, esterified cholesterol solubilization leads to a 100-fold increase in DENV genome copies in the presence of *w*MelPop (59).

We propose that although cholesterol is essential for viral entry, vesicle trafficking, viral assembly, and secretion (70), amino acid consumption by *Wolbachia* is crucial for blocking viral synthesis at the intracellular level. This is consistent with findings made by Caragata et al. (61), where sterol supplementation was not able to completely eliminate the protective effect of this endosymbiont. We have found that the addition of cholesterol to the stoichiometric composition of the simulated arboviral particle has no significant effects on viral replication inhibition by *Wolbachia*.

This is a significant contribution toward unveiling the mechanism of *Wolbachia*-derived pathogen blocking.

### Conclusions.

In this work, we have presented a novel approach for studying *Wolbachia*'s metabolic interaction with Aedes aegypti. By shifting the paradigm of analysis of metabolic networks from the focus on known reactions to identifying gaps as potential interaction points between the endosymbiont and its host, we have developed a methodology that allows us to propose effective coupling points between the metabolic networks of both organisms.

Based on this new methodology, we have developed the first eukaryote-bacterium endosymbiotic genome-scale model. By means of this model, we were able to discover and study key metabolites that must be trafficked between both organisms in order to sustain *Wolbachia* while providing its host with pathogen-blocking capabilities.

Due to its endosymbiotic nature, *Wolbachia* metabolism is characterized by incomplete pathways. On the other hand, *Wolbachia*-infected A. aegypti cells exhibit an altered amino acid and lipid metabolism caused by the survival requirements of the endosymbiont. Associated with this altered lipid metabolic profile, we propose that *Wolbachia* uses cholesterol instead of lipid A as a main membrane component, which is consistent with reported results on perturbed cholesterol metabolism observed in infected mosquitoes.

*Wolbachia*-mediated pathogen blocking has been successfully used as a strategy for disease control, which has motivated an increasing number of studies regarding the underlying mechanisms that result in the pathogen-blocking phenotype. One of the widely accepted conclusions is that common effects between both arboviral and *Wolbachia* effects on host cellular metabolism, such as induced stress as a consequence of depletion of intracellular metabolites and highly dependency of cholesterol for their replication, are key to understanding this complex process.

Based on our analysis using our novel systems biology approach, pathogen blocking was associated with competition for resources, mainly amino acids, cholesterol, and lipids. While cholesterol is essential for viral entry, assembly, and secretion, amino acids are key for blocking viral synthesis at the intracellular level, since our results demonstrate that virus production is highly affected by amino acid availability and consumption. A more detailed sampling approach study showed that competition for protein-derived resources results in high pathogen blocking capabilities at higher *Wolbachia* titers.

This novel approach for studying endosymbiotic systems has allowed us to understand the metabolic interactions between *Wolbachia* and its host and to improve knowledge on the relevant metabolites involved in its survival and pathogen-blocking capabilities, in agreement with previously reported experimental results. We believe that this new paradigm for the analysis of coupled metabolic networks holds the key for unveiling the mechanisms behind complex symbiotic relationships.

## MATERIALS AND METHODS

### Aedes aegypti genome-scale model.

A general metabolic reconstruction for Aedes aegypti was obtained using Pantograph (71) with the human genome-scale model Recon 2.2 (7,785 reactions, 1,675 genes) (19) as a template. Ortholog determination among A. aegypti and Homo sapiens genes was achieved using the inParanoid standalone program (19) with the proteome of A. aegypti (72) and Homo sapiens as input. Using BLOSUM62 as the substitution matrix, with a confidence value cutoff of 0.05 and a group overlap cutoff of 0.5. The obtained orthologs were processed to establish a link between metabolic features of the template model and the target organism.

The obtained biomass function was modified to represent the differences between the human lipid distribution and the one reported for the Aedes albopictus cells growing *in vitro* (73), RNA and DNA composition reported for the insect cell line Sf9 (74), and the distribution of lipids in Aedes aegypti cells cultivated *in vitro* (75). Additional specific metabolic reactions for Aedes aegypti were added based on literature. Validation was performed to test the capability of the model to reproduce growth conditions observed experimentally as well as the effect of gene-knockouts directly associated with metabolic reactions.

### Wolbachia pipientis genome-scale model.

Draft genome-scale models for two Wolbachia pipientis strains (*w*Mel and *w*MelPop) were generated using modelSEED (13). The obtained metabolic networks were analyzed individually to determine which absent metabolic reactions correspond to metabolic gaps using blast. Matches in *Wolbachia* genomes for strains *w*Mel and *w*MelPop with a coverage value above 85% and E values lower than 0.001 were considered metabolic functionalities preserved in this endosymbiont. Biomass determination was constructed using Escherichia coli (76) composition as a template with modifications based on experimental data for phospholipid composition of Rickettsia prowazekii (17, 18). Computation of the stoichiometric coefficients associated with each component in the biomass reaction was done as it has been previously described by Thiele et al. (10). The curated models for both strains were combined using COBRApy (77), preserving gene association rules for both strains in order to generate a Wolbachia pipientis genome-scale model.

### *Wolbachia*-mosquito symbiotic model.

Both models were combined to obtain a *Wolbachia*-mosquito model in which *Wolbachia* is represented as an intracellular compartment of Aedes aegypti. *Wolbachia* transport reactions were modified to move metabolites toward the cytoplasm instead of the extracellular compartment of the global model while exchange reactions were deleted, since *Wolbachia* should get all their resources from the mosquito metabolic network.

*Wolbachia* exchange reactions associated with metabolites absent in the Aedes aegypti network are conserved and considered to be delivered directly into *Wolbachia*. The obtained model was checked using memote (78) and subjected to flux balance analysis (FBA) tests to ensure that this model is able to produce biomass for both Aedes aegypti and Wolbachia pipientis, using the maximization of mosquito biomass as an objective function subjected to a minimum of *Wolbachia* growth as a constraint.

### Viral replication.

A viral production reaction that comprises nucleotide, amino acid, and lipid composition of the Dengue virus is included in the symbiotic model. Nucleotide and amino acid stoichiometric representation was obtained from Aller et al. (65), lipid composition and weight fraction were derived from Reddy and Sansom (64), which was transformed into stoichiometric coefficients, as previously described in Thiele and Palsson (10).

### Simulations.

FBA for each organism was achieved using cell growth as their optimization objective. An additional constraint derived from the reported number of *Wolbachia* organisms inside a mosquito cell was imposed in the symbiotic system (60), where maximization of Aedes aegypti's biomass synthesis is the optimization objective of the whole system.

In order to study *Wolbachia*'s pathogen blocking properties, FBA was performed in a virus-infected Aedes aegypti cell with and without *Wolbachia*, imposing virus replication as maximization objective with a fixed Aedes aegypti cell replication.

Sampling of the solution space was achieved by using the sampler included in COBRApy (77). This sampler performs a uniform random sample of the solution space to study *Wolbachia*'s metabolism without imposing an objective function.

### Data availability.

Supporting information is available to download at https://github.com/natJimenez/symbioticModelAnalysis.
